# Cardiac Syncope: An Underestimated Cause of Unexplained Syncope in the Elderly-Data from a Single High-Volume Syncope Unit

**DOI:** 10.3390/jcm15062450

**Published:** 2026-03-23

**Authors:** Stefanos Archontakis, Evangelos Oikonomou, Nikias Milaras, Panagiotis Dourvas, Tzonatan Klogkeri, Dimitrios Kalantzis, Anastasios Markakos, Michail Ampeliotis, Artemis Papadima, Dimitrios Venetsanos, Sotirios Tsalamandris, Dimitrios Syrseloudis, Skevos Sideris

**Affiliations:** 1 Department of Cardiology, Hippokration General Hospital, 114 Vasilisis Sofias Str., 115 27 Athens, Greece; 2 Third Cardiology Division, Medical School, Sotiria Thoracic Diseases Hospital, University of Athens, 152 Mesogeion Ave., 115 27 Athens, Greece; 3 Department of Cardiac Surgery, Hippokration General Hospital, 114 Vasilisis Sofias Str., 115 27 Athens, Greece

**Keywords:** Syncope Unit, implantable loop recorder, unexplained syncope, cardiogeriatric assessment, elderly patients

## Abstract

**Background/Objectives**: Syncope remains a common problem in the elderly, adversely affecting quality of life, morbidity and mortality. Diagnosis is challenging due to the atypical presentation, multifactorial aetiology, overlap with non-syncoptic falls and increased prevalence of cardiac disease. This study aims to investigate the impact of cardiac syncope in this high-risk population. **Methods**: A retrospective single-centre observational cohort study, including 171 patients ≥65 years old with syncope of unknown origin or other falls, was conducted. Different diagnostic tests and strategies were utilised during the investigational process, based on clinical judgement and the latest guidelines. Patients were classified either in the ‘high risk’ (‘cardiac’) or ‘low-risk’ (‘autonomic’) pathway. **Results**: Mean age was 76.4 ± 6.6 years (range: 65–92 years old) and the mean follow-up period was 40.5 months. Our study population was characterised by a high incidence of comorbidities and underlying heart disease, and polypharmacy. One third of the patients did not report prodromals, 81.9% had no recognisable trigger and 43.3% had various 12-lead ECG abnormalities. Overall, 67.8% of the patients were stratified in the ‘cardiac pathway’. Eventually, a final diagnosis was established in 126 patients (73.7%). The cause was cardiac syncope in 56.4%, reflex syncope in 26.2%, orthostatic hypotension in 7.9% and non-syncopal falls in 9.5%. An ILR was implanted in 90.1% with a diagnostic yield of 43%. ECG-based diagnosis occurred in 53.2% whereas time to diagnosis was 4.8 ± 3.3 months. **Conclusions**: Cardiac disease, mostly arrythmias, represent a common and possibly underestimated cause of unexplained syncope in the elderly. A structured approach including a targeted use of ILRs improves investigational process.

## 1. Introduction

According to recent estimates, the population of elderly people (usually defined as those over 65 years old) in Europe is expected to increase significantly, rising from 90.5 million in 2019 (20.3% of the population) to 129.8 million (nearly 30% of the population) by 2050, while those aged 75–84 years are expected to expand by 56.1% [1]. Syncope, on the other hand, defined as a self-limited transient loss of consciousness and postural tone due to rapid onset global cerebral hypoperfusion with a short duration and complete recovery, has a higher incidence in the elderly than in any other age group [2,3]. The incidence of syncope is significantly increasing from 5.7 episodes/1000 individuals per year between 60 and 69 years old to 11.1 episodes/1000 individuals per year between 70 and 79 years, showing a further increase to 19.5 episodes per 1000 individuals in the age group over 80 years old [3,4]. Importantly, in adults over 65, syncope is associated with increased mortality, irrespective of the cause [5]. In addition, syncope is a major cause of morbidity. Hospitalisations occur relatively more often in older patients, with 58% of sufferers over 80 years being admitted to hospital, and are associated with increased health economic costs [6,7]. Moreover, syncope and the subsequent fear of falling has a particularly adverse impact on the quality of life (QoL) of older adults [8]. Finally, injurious events such as fractures and head injuries are also more common in this patient group [9].

In contrast to younger patients, syncope in the elderly is often characterised by atypical presentation. Patients often do not report prodromes and may have amnesia for the events. History is often unreliable and frequently unwitnessed and therefore there might be an overlap with drops. Moreover, syncoptic episodes in this patient group are usually multifactorial and may be associated with many predisposing factors. Polypharmacy and the presence of comorbidities are also frequent [7,8,9].

Diagnosis and treatment of syncope in older patients is challenging, due to the atypical presentations, the wide range of differential diagnosis, polypharmacy, the increased incidence of comorbidities, and the physiological changes associated with ageing. Advanced clinical skills and expertise are required to reduce hospital admissions and improve quality of life in this increasingly complex population.

We have observed that cardiac causes and syncope attributed to more than one possible cause are common in the clinical practice in older subjects with unexplained syncope assessed in our Syncope Unit, which is based in the cardiology clinic of a tertiary hospital. Therefore, we speculate that cardiac arrhythmias are an underdiagnosed cause of syncope of unknown origin. The objective of this study is to determine the incidence of cardiac syncope, particularly due to arrhythmias, in the elderly population in the era of modern algorithms for diagnostic investigation and an extended albeit targeted, use of implantable cardiac loop recorders (ILRs).

## 2. Methods

### 2.1. Study Design and Patient Population

A single-centre, retrospective, observational cohort study undertaken in a tertiary Syncope Unit was conducted. Individuals ≥ 65 years old with syncope of unknown origin or other possible falls admitted in our clinic from March 2019 until December 2024 were recruited in the study.

The Syncope Unit is located at the Cardiology Department of Hippokration Hospital, Athens, Greece, operating mainly as an outpatient clinic in line with the European Society of Cardiology (ESC) guidelines [2,10]. In addition, patients admitted in the hospital may also be reviewed if requested by the treating physicians. Outpatient referrals for further assessment and consulting originate either from the various outpatient departments of our hospital or other hospitals, or from the emergency department; however, the possibility of self-referral is also offered.

In addition to age ≥ 65 years old, the study inclusion criteria were: (a) individuals with presyncope or syncope episodes [2] assessed in our clinic; (b) individuals with episodes of TLOC [2] assessed in our clinic; (c) individuals referred from other facilities for episodes of ‘unexplained syncope’ (US) or unexplained ‘falls’; and (d) absence of severe comorbidities or systematic diseases limiting prognosis to less than 1 year.

All patients presented in our facility were investigated according to the current European Society of Cardiology (ESC) guidelines [2]. Firstly, individuals were assessed according to the ‘initial evaluation’ scheme, including an extensive history of both the conditions of occurrence of the syncoptic episode, any additional health problems and the patient’s medication list. Initial evaluation also included a detailed clinical examination, performing an active standing test and a carotid sinus massage when clinically relevant, and a 12-lead electrocardiogram (ECG). Patient data were stored in both electronic and paper forms.

Assessment scheme was subsequently individualised based on clinical judgement and subjects were recruited either in the ‘cardiac’ (‘high-risk’) or ‘autonomic’ (‘low-risk’) pathways. Stratification process followed the ESC guidelines [2]. The presence of a high probability for arrhythmia as the underlying mechanism in combination with the presence of ECG abnormalities guided our decision for selecting the most adequate test for further investigating syncope. Novel algorithms based on up-to-date research were followed for patients categorised in the ‘cardiac’ and ‘autonomic’ pathways [11,12]. In all cases, 24 h ECG ambulatory monitoring and blood tests including full blood count, kidney function tests and electrolytes and hepatic function tests as well as a transthoracic echocardiogram were performed. Despite its emerging significance, 24 h ambulatory blood pressure monitoring (24 hABPM) was underutilised in our practice.

Further assessment was performed according to clinical judgement and included tilt table test (TTT), electrophysiological study (EPS), and/or long-term ECG recording with an ILR. For TTT, either the standard 20 min Italian Protocol or the new ‘fast’ Italian protocol was selected, including provocation by means of nitrogen triglyceride sublingual spray. A functional ischaemia test (e.g., Treadmill Test, Stress echocardiography or Scintigraphy) in order to investigate the presence of myocardial ischaemia and to assess possible exercise or post-exercise syncope was considered after case-by-case assessment. A coronary angiogram was programmed in the case of an increased possibility of ischaemia. Depending on the patient history, referrals to internal medicine or neurology clinics were possible.

In those patients in whom a Loop Recorder was favoured, implantation of a Reveal LINK^TM^ or a LINK II ^TM^ Insertable Cardiac Monitoring System (Medtronic Inc., Minneapolis, MN, USA) occurred. Remote Monitoring (CARELINK^TM^ NETWORK, Medtronic Inc., Minneapolis, MN, USA) was utilised for remote day-by-day assessment. Arrhythmias recorded by the device were classified according to the ISSUE study criteria [13]. Patient triggered events, marked as ‘symptoms’, were recorded and after contacting the patient, the nature of the symptom was assessed. Patients were also advised to contact the centre in the presence of unexplained symptoms. ECG diagnosis occurred in the case that an arrhythmia was recorded in combination with clinically relevant symptoms or when serious arrhythmias occurred. Arrhythmias not considered relevant to the investigation of syncope were also recorded.

Follow-up visits in our facility were programmed depending on the clinical condition of the patient. During follow-up assessment, history was obtained and clinical examination was performed, and the investigation scheme was revised. Unscheduled visits could also occur accordingly. On the other hand, urgent, or elective in hospital admissions occurred according to the clinical findings. Follow-up was performed for at least 12 months after initial assessment in all subjects.

The primary endpoint of the study was the definite diagnosis of syncope. Secondary endpoints were (a) ECG-based diagnosis, (b) the initial presentation to final diagnosis time, (c) incidence of syncope relapse.

Due to the retrospective nature of the study, all eligible patients presenting to the Syncope Unit during the study period were included. No formal sample size calculation was performed.

### 2.2. Ethical Considerations

This study was conducted in compliance with the Declaration of Helsinki and the ethical guidelines for research involving human subjects. The study design was reviewed and approved by the Institutional Review Board (IRB)/Ethics Committee of our hospital (Number 123/4b 18-12-2025). Ethical approval was not considered necessary since the study included only anonymised data from patient medical records managed according to our standard practice without any intervention. Personal data were stored in electronic and paper forms and were pseudonymised. Informed consent for research purposes was not obtained due to the fact that all patients were treated according to standard clinical practice, there was no interventional group and that our study included only retrospective data. However, all patients were verbally informed that their anonymised data may be used for research purposes.

### 2.3. Statistical Analysis

Data are presented as absolute numbers and percentages if categorical and as mean ± standard deviation if continuous. All variables were tested for normality of distribution with p = plots and Kolmogorov–Smirnov test. For categorical variables, differences between different studied groups were tested with Chi-squared test or Fisher’s exact test as appropriate and for continuous variables, with analysis of Variance (ANOVA, New Providence, NJ, USA). Statistical calculations were performed in SPSS software (version 27.0; SPSS Inc., Chicago, IL, USA) and GraphPad Prism (version 8, GraphPad, Boston, MA, USA).

## 3. Results

### 3.1. Study Population

During a 70-month period, from 1 March 2019 until 31 December 2024, a total of 171 patients over 65 years old were referred to our Syncope Unit. The mean age of this population was 76.4 ± 6.6 years (ranging 65–92 years old) and 81 individuals (47.4%) were female. Sixty-two patients were older than 80 years (36.3%). Subjects were referred from the emergency department in 20 cases (11.7%), from our outpatient clinic in 48 cases (28.1%), from other hospitals and primary care structures in 92 cases (53.8%), whereas self-referrals occurred in 11 cases (6.4%). Mean follow-up period was 40.5 months (ranging from 12 to 70 months) (Table 1). Patient demographic data, clinical characteristics and investigational strategies are presented in Table 1 and Table 2.

The study population was characterised by a high burden of comorbidities and cardiovascular disease. Detailed clinical characteristics, comorbidities, and medication use are summarised in Table 1. Polypharmacy was present, as only 7 of the 171 patients did not receive any medication at all. Interestingly, the incidence of patients receiving medication affecting coagulation (i.e., either antiplatelets or anticoagulants) was 39.2%.

Presentation without prodromes was not rare; prodromes were recorded in 114 patients, while one third (n = 57) reported none. Most patients (81.9%) experienced syncope without a recognisable trigger. Syncope while supine or sitting was also common (9.9% and 53.2%, respectively), and palpitations were reported in 14.6%. Elderly patients showed a high incidence of injury (74.3%), including head trauma and limb fractures. Electrocardiographic abnormalities were present in 43.3% of patients.

### 3.2. Initial Evaluation and Diagnostic Investigations

All patients underwent an initial evaluation including detailed clinical history, physical examination, active standing test, and 12-lead electrocardiogram. Orthostatic hypotension was detected during the active standing test in 21 patients (12.3%), although none experienced symptoms during the test. Carotid sinus massage was performed in 58 patients (33.9%), revealing carotid sinus hypersensitivity in 12 cases without reproduction of symptoms.

Additional diagnostic investigations performed during the evaluation process are presented in Table 2. Ambulatory 24 h ECG monitoring was performed in all patients; it was normal in 68 individuals (39.8%) and showed minor, non-diagnostic findings in the remaining 103. These commonly included atrial or ventricular extrasystoles, short runs of supraventricular or non-sustained ventricular tachycardia, and low mean heart rate. Twenty-four patients (14%) were hospitalised for continuous monitoring without significant findings. Transthoracic echocardiography was performed in all 171 patients to evaluate structural heart disease. Although it was not diagnostic for syncope in any case, it guided subsequent diagnostic steps.

**Figure 3:**
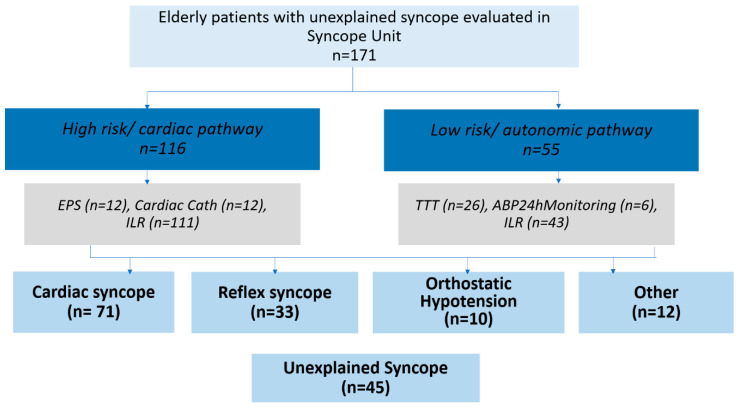
Flow chart of the study.

More than two thirds of the cohort (n = 116, 67.8%) were classified as high-risk and entered the cardiac diagnostic pathway, while the remaining 55 were considered low-risk and followed the autonomic pathway (Figure 3). Tilt table testing was performed in 26 patients (15.2%) and was diagnostic in 11, revealing cardioinhibitory response in one, vasodepressor response in six, and mixed response in four patients. Typical symptoms were reproduced in all diagnostic cases. Electrophysiological study (EPS) was performed in 13 patients (7.6%). Abnormalities included HV interval >70 ms, prolonged corrected sinus node recovery time, and early atrioventricular block during rapid atrial pacing. Programmed ventricular stimulation induced ventricular tachycardia in three patients with ischemic heart disease. In two patients with bifascicular block, marked HV prolongation and early 2:1 atrioventricular block were induced during pacing; one also had prolonged cSNRT with post-pacing pauses.

### 3.3. Implantable Loop Recorder Findings

Implantable loop recorder (ILR) implantation was performed in 154 patients (90.1%). No procedure-related complications occurred. An ILR-based diagnosis was established in 66 patients, typically during recurrence of syncope or presyncope. Among these, tachyarrhythmias were recorded in nine patients (atrial fibrillation in six, supraventricular tachycardia in one, and ventricular tachycardia in two), while bradyarrhythmias were documented in 57 patients (conduction disorders in 32 and sinus arrest in 25).

In an additional 42 patients, ILR findings contributed substantially to the diagnostic process. Symptom reproduction without electrocardiographic abnormalities was recorded in 22 patients with clinical features consistent with reflex syncope. In 10 patients, symptoms occurred during transition to the upright position and were associated with significant blood pressure reduction during active standing, suggesting orthostatic hypotension. Eight patients experienced falls during standing or walking without electrocardiographic abnormalities or significant hypotension, consistent with drop attacks. One patient was diagnosed with epilepsy after an episode of loss of consciousness with myoclonus and normal ILR recordings. Another patient with dizziness and fall was found to have severe hyponatraemia and hypokalaemia despite electrocardiographic abnormalities recorded by the ILR.

### 3.4. Primary Endpoint

**Figure 1:**
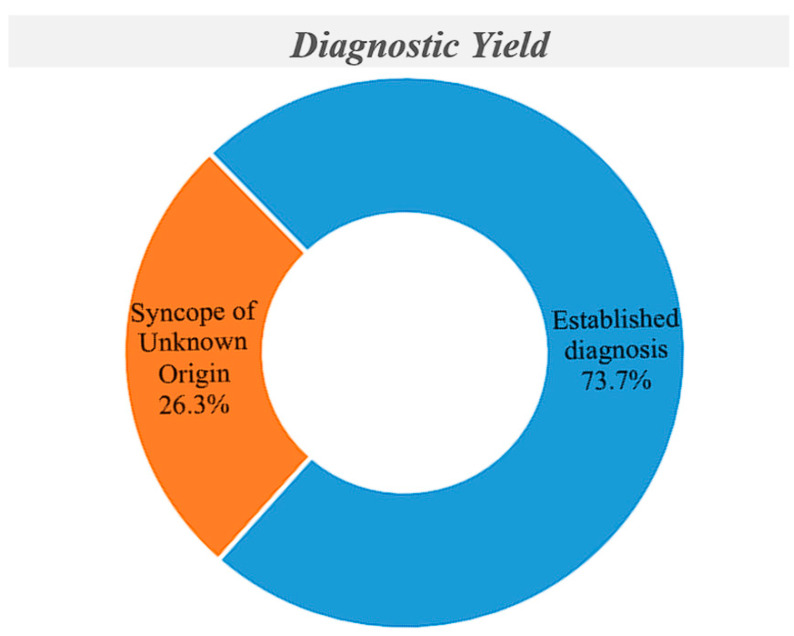
Diagnostic yield of the investigational process followed in the Syncope Unit in the elderly population, ≥65 years old.

**Figure 2:**
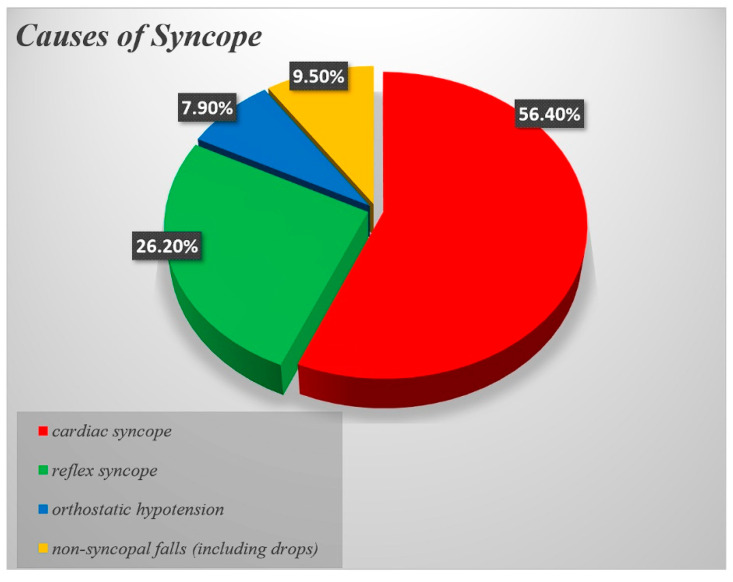
Causes of syncope in the elderly population (i.e., ≥65 years old) of the Syncope Unit.

Minimum follow-up was 12 months (range 12–82), with a mean of 40.5 ± 20 months. A final diagnosis was established in 126 of 171 patients (73.7%), while in 45 individuals the cause remained unexplained (Table 3, Figure 1). Cardiac syncope was diagnosed in 71 of the 126 patients (56.4%), based on ILR findings (n = 66) or EPS (n = 5). Vasovagal reflex syncope was diagnosed in 33 patients (26.2%) using history, tilt testing (n = 11), and ILR findings (n = 22). Orthostatic hypotension was considered the cause in 10 patients (7.9%) based on clinical history, standing test results, and exclusion of arrhythmic causes. Non-syncopal causes were identified in 12 patients (9.5%), including eight drop attacks, two epilepsy cases, one case of persistent hyponatraemia with hypokalaemia, and one pulmonary embolism. All patients with unexplained syncope received an ILR (Figure 2, Figure 3, Table 3).

### 3.5. Secondary Endpoints

The most common mechanism of cardiac syncope was bradycardia, including long sinus pauses suggesting sick sinus syndrome or pauses due to atrioventricular block. An ECG-based diagnosis using loop recorder recordings was achieved in 66 of 71 patients with cardiac syncope. Additionally, one cardioinhibitory event recorded on ILR confirmed reflex syncope. Overall, an ECG-based diagnosis occurred in 67 of 126 patients (53.2%) (Table 3, Figure 3).

Time to diagnosis varied, but the final diagnosis was established on average 4.8 months after the first visit to the Syncope Unit (Table 3).

During a mean follow-up of 40.5 months, syncope recurrence occurred in 8 of 71 patients with cardiac syncope (11.6%), 12 of 33 with reflex syncope (36.4%), and 4 of 10 with orthostatic hypotension (40%).

Among the 154 patients with ILR, various non-diagnostic arrhythmias were recorded, including frequent premature ventricular contractions (>5%), atrial fibrillation, supraventricular arrhythmias, asymptomatic bradycardia < 40 bpm, and isolated non-conducted P waves.

### 3.6. Therapy

Among 59 patients with bradycardia-related syncope, 54 received pacemakers, while five declined implantation and remained under close follow-up with medication adjustment. Symptomatic atrial fibrillation with rapid ventricular response occurred in six cases and was treated by ablation in one and medically in five using β-blockers, diltiazem, class Ic anti-arrhythmics, or combinations. The ablation case relapsed and was subsequently treated with pacemaker implantation and atrioventricular junction ablation.

One patient with supraventricular tachycardia underwent electrophysiological study and AVNRT ablation. Ventricular tachycardia was recorded in five patients: three ischemic patients during EPS, one ischemic patient with ILR, and one patient with hypertrophic cardiomyopathy. Three patients received implantable cardioverter defibrillators (ICDs), while two received biventricular defibrillators due to moderate left ventricular dysfunction and conduction disease.

Of the 33 patients with reflex syncope, one received a pacemaker and 32 were treated conservatively. Orthostatic hypotension was managed conservatively without medication. Patients with non-syncopal causes were treated accordingly. Finally, four additional patients were diagnosed with paroxysmal atrial fibrillation and started on anticoagulation, although the arrhythmia was considered unrelated to syncope.

## 4. Discussion

In this retrospective single-centre observational study of elderly patients referred to a tertiary Syncope Unit for evaluation of unexplained syncope or falls, several important findings emerged. First, most patients were classified as high-risk and entered the cardiac diagnostic pathway. Second, a final diagnosis was established in nearly three quarters of the study population, with cardiac syncope representing the most frequent cause. Third, prolonged rhythm monitoring with implantable loop recorders played a pivotal role in the diagnostic process, providing an ECG-based diagnosis in a substantial proportion of patients and contributing to the exclusion of arrhythmic causes in others. Finally, the implementation of a structured diagnostic algorithm within a dedicated Syncope Unit resulted in a relatively short time to diagnosis and a low recurrence rate of syncope during follow-up. Collectively, these findings suggest that cardiac causes, particularly arrhythmias, may represent an underrecognised mechanism of unexplained syncope in the elderly and highlight the importance of systematic evaluation and prolonged cardiac monitoring in this high-risk population.

Syncope remains a common problem that has a particularly negative impact on patients’ prognosis and quality of life in the elderly. Our data revealed a high incidence of injury of 74.3% in the patients who visited our facility and were over 65-year-old, including head and limb trauma. In addition, syncope represents a challenging clinical entity, due to the wide spectrum of possible causes that could be both benign and potentially life-threatening. The combination of history taking and physical examination, and 12-lead ECG remains the cornerstone for diagnosis [2]. Following initial evaluation, further diagnostic work-up of the still unexplained syncoptic episode is based on the classification either to the ‘cardiac’ or ‘autonomic’ pathway, utilising a risk stratification algorithm based on clinical judgement. In patients presenting with high-risk features, suggesting cardiac syncope, ‘cardiac tests’ should be performed as the first step [11]. On the other hand, low-risk patients in whom autonomic, non-cardiac syncope is speculated should be further investigated in order to identify whether a hypotensive or bradycardic phenotype is responsible for syncope [11,12]. Prolonged ECG monitoring by means of an implantable loop recorder remains the final step of both cardiac and autonomic pathways when the cause of syncope remains unexplained [11]. Furthermore, several studies provide evidence highlighting the importance of dedicated Syncope Units and loop recorders for improving diagnostic yield [10,14,15,16].

In our facility, we intend to be as strict as possible in following the above-described strategy, irrespective of the age of the patient. According to our data, the vast majority (i.e., 116 of 171, 67.8%) of the elderly patients, aged ≥65 years, assessed in our Syncope Unit were stratified in the ‘cardiac’ (high-risk) pathway due to the frequent lack of prodromes or triggers, inadequacy of providing history, amnesia, syncope at supine or sitting position or during exercise, presence of preexisting heart disease, presence of symptoms such as palpitations and presentation with electrocardiographic abnormalities. These patients underwent various tests such as cardiac catheterisation (n = 12, 7.2%), non-invasive stress tests (n = 42, 24.6%) and electrophysiology study (n = 13, 7.6%). The remaining 55 patients (32.2%) were stratified in the autonomic pathway, undergoing tilt table test in 26 cases (15.2%), whereas 24 h blood pressure monitoring was underutilised (n = 6, 3.5%). Irrespective of the pathway in which the patient was classified, standing test (100% of patients), carotid massage test (33.9% of patients), echocardiogram (100% of patients) and 24 h ambulatory electrocardiography (100% of patients) were also performed.

An additional finding from our study is the high likeliness of utilising a loop recorder in this age group. In our study group, 154 of the 171 patients (90.1%) eventually underwent ILR implantation. Similarly to our data, earlier studies had shown that early insertion of an implantable loop recorder is more likely to occur in older subjects and that the ILR, most probably, has a higher diagnostic value in this population [17,18]. In this study the diagnostic yield of the ILR intervention, defined as an ILR-based electrocardiographic diagnosis, was 43% (66 of the overall 154 patients in whom it was implanted). However, importantly, ILR implantation may have an even greater impact and further assist diagnosis by excluding arrythmias in symptomatic patients in whom the ECG is normal during a typical syncoptic event. Of note, in the present study in an additional 42 patients the ILR critically assisted diagnosis by excluding arrhythmic syncope. Overall, in line with other studies [17,19], our data demonstrate a high diagnostic yield of ILR in elderly patients with unexplained syncope and support assessment with continuous prolonged cardiac monitoring in this population to detect underlying cardiac arrhythmias. Therefore, we may conclude that implantation of an ILR is a safe and effective measure in the investigation of syncope in the elderly population.

Third, an electrophysiological study was performed in 13 elderly patients (7.6%). Although several studies have questioned the role of EPS showing a moderate negative predictive value, other authors showed that abnormal ECG findings during non-invasive testing are well correlated with pathognomonic findings in EPS, suggesting arrhythmic causes in patients with undiagnosed syncope [20,21]. Based on our data, we conclude that EPS remains a useful tool, in selected cases, that could potentially assist the investigational process.

Another significant finding from our work is that adoption of a strict algorithmic strategy during investigation in the Syncope Unit may lead to a high diagnostic yield in this age group. In our study, a final diagnosis was achieved in 73.7% of the admitted patients. Moreover, the final diagnosis was cardiac syncope in 56.4% of those patients in whom the investigational process was successful. Therefore, a major finding in this study is that cardiac causes are relatively more common as patients age.

Similarly to other age groups, in the elderly population syncope may be classified as follows [22].

(a) *Reflex syncope*, including vasovagal syncope and carotid sinus syndrome, is considered to be the most common type of syncope in older adults. In these patients, due to the reduction in the vagal tone, vasodepressor syncope is more common, often occurring without prodromals. In addition, vasovagal syncope may occur under conditions associated with a sudden change in the autonomic tone such as micturition, defecation, exercise, or coughing. Moreover, carotid sinus syndrome is increasingly common at older ages where stiffness of the carotid vasculature is increased due to atherosclerosis and ageing.
(b) *Orthostatic hypotension* is also common in the elderly, occurring in up to 30% among patients older than 75 years old. Underlying autonomic insufficiency, either idiopathic or resulting from comorbid conditions such as diabetes, amyloidosis, or neurological disorders, should be considered in older adults, especially in recurrent syncope. Postprandial syncope (defined as syncope that occurs following a meal) is a common subtype of orthostatic hypotension, attributed to venous pooling in the splanchnic vessels, resulting in reduction in the effective blood volume.
(c) *Cardiac syncope*, due to arrhythmias or cardiac structural diseases (e.g., aortic stenosis), is considered to be a less common cause of syncope in older adults compared to reflex syncope; however, it is associated with worse outcomes. The prevalence of bradycardias is increasing with age and may result from medications, sick sinus syndrome, or atrioventricular block, whereas atrial and ventricular tachycardias may also result in syncope through a reduction in stroke volume as a result of incomplete myocardial relaxation and filling.

Previous studies have demonstrated that despite the high incidence of heart disease in the elderly, cardiac syncope does not show increase with age relative to other causes [7]. In contrast to this perception, in our study, in most of the patients in whom a final diagnosis was achieved, the cause was cardiac syncope (56.4%). Moreover, reflex syncope, orthostatic hypotension and non-syncopal falls (including drops) were the causes in 26.2%, 7.9% and 9.5% of the patients, respectively (Table 3). Our data suggest that cardiac syncope may be more frequent than previously believed in the elderly population presenting with unexplained syncope. However, we should also appreciate that there is an inhomogeneity in the investigational process of syncope among different health systems. Most of our patients were referred to us at a later stage, having already been investigated in other centres. Therefore, the study population in our facility may not be necessarily a representative sample of the general population although it is indeed a representative sample of the type of patients referred for further specialised assessment. Our Syncope Unit operates as a tertiary centre, and therefore more obvious aetiologies, such as carotid sinus syndrome and orthostatic hypotension, may have been identified prior to referral.

An overlap between syncope and drops in older individuals is assumed since amnesia of the event and lack of witnessing the ‘fall’ event is very common [19]. In the older patients of our study population, mostly in those over 80 years old, difficulties in obtaining the history of the event were significant. We speculate that a significant subset of the individuals in whom the aetiology remained unidentified probably represent patients with drops and not ‘true’ syncope. Nevertheless, in line with other investigators [23], we also believe that differential diagnosis among drops and ‘true’ syncopal causes such as vasovagal syncope, carotid sinus syndrome, orthostatic hypotension and cardiac arrhythmias is challenging if not impossible.

Moreover, we believe that distinguishing between a cardioinhibitory vasovagal reaction and a pause or complete heart block due to sick sinus syndrome or true conduction disorders in the elderly is practically impossible. In most of the cases these findings are characterised as true cardiogenic syncope.

Fifth, our data show that during the mean 40.5 months follow-up, the relapse of syncope in those diagnosed with cardiac syncope was dramatically reduced, indicating that the initial diagnosis was correct. In those diagnosed with reflex syncope or orthostatic hypotension, relapses were also significantly reduced. However, falls did happen in 11.6% of the patients diagnosed with cardiac syncope, a finding that probably indicates the multifactorial mechanism of syncope in elderly patients.

Sixth, recent evidence suggests that sodium–glucose co-transporter 2 inhibitors (SGLT2i) may exert relevant anti-arrhythmic effects in multimorbid patients, potentially reducing the risk of arrhythmia-related events, including cardiac syncope [24]. While this remains hypothesis-generating, it highlights a possible preventive strategy in elderly patients at high risk of cardiac syncope, particularly those with concomitant heart failure or diabetes. Further studies are needed to confirm the clinical benefit of SGLT2i specifically in this population.

Finally, an additional consideration is the relative detectability of different arrhythmias in elderly patients. Our data show that during follow-up by continuous monitoring using ILR, various cardiac arrythmias were detected that were not considered relevant to the cause of the syncoptic episode. However, in some cases, the presence of these arrhythmias resulted in treatment interventions, such as in the case of 4 patients diagnosed with paroxysmal atrial fibrillation who received anticoagulation. Bradyarrhythmias are often persistent and therefore more readily identified during routine evaluation, whereas paroxysmal tachyarrhythmias, particularly atrial fibrillation, may remain undetected without prolonged monitoring. The use of ILRs in our cohort allowed identification of intermittent arrhythmic events that might have otherwise been missed. Consequently, the observed predominance of bradyarrhythmias in our findings may partially reflect the inherent challenges in capturing transient tachyarrhythmias, highlighting the value of extended cardiac monitoring in this population.

Our study adds incremental value to the existing literature on syncope management in several ways. First, we focus on an older, multimorbid population (≥65 years), which is often underrepresented in previous Syncope Unit studies, and in whom diagnostic evaluation is particularly challenging due to atypical presentations, polypharmacy, and comorbidities. Second, we implemented a pathway-based triage distinguishing ‘high-risk’ (cardiac) and ‘low-risk’ (autonomic) patients, reflecting a structured real-world workflow that aligns with guideline recommendations but has not been extensively described in large cohorts of elderly patients. Finally, the high utilisation of ILRs in this setting provides insight into the diagnostic yield and arrhythmic patterns in this population, highlighting practical implications for the management of unexplained syncope in routine clinical practice.

The present study has important limitations [25]. First, it is a single-centre retrospective observational cohort study that has recruited a restricted number of participants. Moreover, patients were referred from different facilities (e.g., primary healthcare, cardiology clinics, other medical specialties). Therefore, there was remarkable inhomogeneity regarding the extent of previous investigations with some patients being more extensively investigated than others. Third, the study population may not be representative of the general elderly population, since referrals occurred in already investigated patients. This may have resulted in over-presentation of cardiac syncope cases. The prevalence of cardiac arrhythmia in this cohort appeared significantly higher; however, the implication of our findings in other settings, such as in the primary care, may not be appropriate. Fourth, despite its emerging significance, 24 h ambulatory blood pressure monitoring was underutilised in our practice. Furthermore, carotid sinus massage was performed in only 58 subjects (33.9%). Sixth, the diagnostic work-up was not fully standardised, and investigations—including the use of implantable loop recorders (ILRs)—were performed at the discretion of the treating physicians based on clinical judgement and guideline-based risk stratification. This may have introduced heterogeneity in the final diagnoses. However, this approach reflects real-world practice in a tertiary Syncope Unit, where individualised assessment is necessary due to the complexity and comorbidity burden of elderly patients. Finally, a comprehensive geriatric assessment as well as a cognitive impairment assessment using validated tools such as the mini-mental state examination (MMSE) was not performed.

## 5. Conclusions

In conclusion, syncope remains a common and clinically important problem in the elderly, often presenting with multifactorial causes. In our single-centre cohort, a structured assessment in a dedicated Syncope Unit, combined with selective use of implantable loop recorders, was associated with a high diagnostic yield and allowed identification of cardiac syncope in a substantial proportion of patients. These findings suggest that cardiac causes may be more frequent than previously appreciated in this population. However, given the retrospective design, single-centre setting, and variability in diagnostic pathways, our results should be interpreted with caution. Further multicentre, prospective studies are warranted to confirm these observations and to optimise strategies for the evaluation and management of unexplained syncope in older adults.

## Figures and Tables

**Table 1:** Patient demographics and clinical characteristics.

Characteristics	Population (n = 171)
Female, n (%)	81 (47.4%)
Age, years	76.4 ± 6.6
**Characteristics of syncope/presyncope**	
Syncope ± presyncope	158 (92.4%)
Presyncope only	13 (7.6%)
Traumatic syncope, n (%)	127 (74.3%)
Νumber of episodes	6.2 ± 3.8
Prodromes ^1^, n (%)	114 (66.7%)
Predisposing factors ^2^	31 (18.1%)
Position, n (%) Supine Sitting Standing Changing	17 (9.9%) 91 (53.2%) 122 (71.3%) 15 (8.8%)
Exertion status, n (%) Rest Mild Intense Post exertion	117 (68.4%) 90 (52.6%) 9 (5.3%) 1 (0.6%)
Presyncope, n (%)	109 (63.7%)
Palpitations, n (%)	25 (14.6%)
Post syncope symptoms, n (%)	141 (82.5%)
Mean recovery time (min)	5.6 ± 4.1
**Clinical Characteristics**	
Arterial Hypertension, n (%)	106 (62%)
Dyslipidaemia, n (%)	95 (55.6%)
Diabetes Mellitus, n (%)	26 (15.2%)
Smoking, n (%)	37 (21.6%)
Coronary Heart Disease, n (%)	30 (17.5%)
Non-Ischaemic Dilated Cardiomyopathy, n (%)	1 (0.6%)
Hypertrophic Cardiomyopathy, n (%)	2 (1.2%)
Atrial Fibrillation ^4^, n (%)	28 (16.4%)
Pacemaker, n (%)	3 (1.8%)
Valvular Heart Disease ^3^, n (%)	11 (6.4%)
Mitral Valve Prolapse, n (%)	3 (1.8%)
AVNRT, n (%)	1 (0.6%)
Previous Stroke, n (%)	6 (3.5%)
Carotid stenosis not requiring operation, n (%)	11 (6.4%)
History of cancer, n (%)	9 (5.3%)
History of thyroid disease, n (%)	33 (19.3%)
History of Parkinson disease on medication, n (%)	6 (3.5%)
History of prostate hyperplasia on medication, n (%)	12 (7.1%)
Receiving medication for dementia, n (%)	14 (8.2%)
Receiving antiepileptic therapy, n (%)	4 (2.3%)
Receiving any chronic medication, n (%)	164 (96%)
Receiving antiplatelet or anticoagulant medication, n (%)	67 (39.2%)
**12-lead ECG abnormalities on presentation, n (%)** (Sinus Bradycardia, 1st degree AV block, Bifascicular block, Atrial Fibrillation, Left Anterior Fascicular Block, Right Bundle Branch Block, Left Bundle Branch Block, n Incomplete Right Bundle Branch Block, Left Ventricular Hypertrophy, Ischaemic Changes, T-wave inversion, Prolonged QT, Early Repolarisation, premature Atrial Contractions, Premature Ventricular Contractions, Paced)	74 (43.3%)

^1^ Prodromes included symptoms such as dizziness, nausea, sweeting, abdominal pain, shortness of breath, palpitations, difficulty in maintaining body stature, tinnitus, etc., or a combination of the above symptoms. ^2^ Predisposing factors included situations such as psychological stress, acute pain, long-standing, postprandial period, post urination/defecation period, standing, hot or crowded environment. ^3^ Valvular heart disease included 5 patients with moderate aortic or mitral regurgitation, 2 patients with bioprosthetic aortic valve, 3 patients with mechanic aortic valve and 1 patient with mechanic mitral valve. ^4^ Atrial fibrillation was permanent in 22 cases and paroxysmal in 6 cases. One patient underwent ablation 8 years prior presentation.

**Table 2:** Additional tests performed after initial evaluation with history, clinical examination and ECG.

Test Performed	Population (n = 171)
Carotid Massage, n (%)	58 (33.9%)
Active Standing, n (%)	171 (100%)
Haematology/Biochemistry, n (%)	171 (100%)
Echocardiogram, n (%)	171 (100%)
Left Ventricular Ejection Fraction	54 ± 10%
ECG Monitoring, n (%)	24 (14%)
24 h ambulatory ECG recording, n (%)	171 (100%)
Treadmill ECG stress test, SPECT or stress echo test, n (%)	42 (24.6%)
Coronary Catheterization, n (%)	12 (7.2%)
Tilt Table Test, n (%)	26 (15.2%)
Electrophysiological Study, n (%)	13 (7.6%)
Implantable Loop Recorder insertion, n (%)	154 (90.1%)
Central Nervous System Computerised Tomography and/or Magnetic Resonance Imaging performed for syncope investigation, n (%)	4 (2.3%)
24 h Ambulatory Blood Pressure Monitoring (24 hABPM), n (%)	6 (3.5%)

**Table 3:** Diagnostic yield and time to diagnosis.

	Population (n = 171)
Mean Follow-up time, m	40.5 ± 20
Established final diagnosis, n (%)	126 (73.7%)
**Cause of syncope, n (%)**	
**Reflex** *Cardioinhibitory*	**33 (26.2%)** *1 (3%)* ^ 1^
**Cardiac** *Bradycardia* *Tachycardia* • *AF* • *SVT* • *VT*	**71 (56.4%)** *59 (83.1%)* ^2^ *6 (8.5%)* ^2^ *1 (1.4%)* ^2^ *5 (7.0%)* ^2^
**Orthostatic Hypotension**	**10 (7.9%)**
**Other (Non-syncoptic)** ^3^	**12 (9.5%)**
Established ECG-based diagnosis, n (%)	67 (53.2%)
Time to final-diagnosis, months	4.8 ± 3.3

*AF: Atrial fibrillation; SVT: supraventricular tachycardia VT: ventricular tachycardia. *^1^ Calculated as a proportion of reflex syncope. ^2^ Calculated as a proportion of cardiac syncope. ^3^ Other causes included: 8 patients with drops, 2 patients with epilepsy, 1 patient with persistent hyponatraemia and hypokalaemia and 1 patient with pulmonary embolism.

## Data Availability

Data will be provided on request at email stef6arch@yahoo.com.
